# Do acute psychological and psychobiological responses to trauma predict subsequent symptom severities of PTSD and depression?

**DOI:** 10.1016/j.psychres.2007.08.014

**Published:** 2008-10-30

**Authors:** Thomas Ehring, Anke Ehlers, Anthony J. Cleare, Edward Glucksman

**Affiliations:** a Department of Psychology, Institute of Psychiatry, King's College London, De Crespigny Park, London SE5 8AF, UK; b Department of Psychological Medicine, Institute of Psychiatry, King’s College London, De Crespigny Park, London SE5 8AF, UK; c King's College Hospital, Denmark Hill, London SE5 9RS, UK

**Keywords:** Cortisol, Cardiovascular arousal, Cognitive processing, Posttraumatic stress disorder, Depression, Predictors

## Abstract

The study investigated the relationship between the acute psychological and psychobiological trauma response and the subsequent development of posttraumatic stress disorder (PTSD) and depressive symptoms in 53 accident survivors attending an emergency department. Lower levels of salivary cortisol measured in the emergency room predicted greater symptom levels of PTSD and depression 6 months later, and lower diastolic blood pressure, past emotional problems, greater dissociation and data-driven processing predicted greater PTSD symptoms. Heart rate was not predictive. Low cortisol levels correlated with data-driven processing during the accident, and, in female participants only, with prior trauma and prior emotional problems. Higher evening cortisol 6 months after the accident correlated with PTSD and depressive symptoms at 6 months, but this relationship was no longer significant when levels of pain were controlled. The results support the role of the acute response to trauma in the development and maintenance of PTSD and provide promising preliminary evidence for a meaningful relationship between psychobiological and psychological factors in the acute trauma phase.

## 1 Introduction

Post-traumatic stress disorder (PTSD) is associated with several psychobiological alterations (for reviews see Olff et al., 2005; Vasterling and Brewin, 2005), but it remains unclear whether these changes are risk factors or consequences of PTSD because most studies used a cross-sectional design. Relatively few studies to date have investigated whether psychobiological variables in the immediate aftermath of trauma *predict* subsequent PTSD.

There is preliminary evidence suggesting that *low* cortisol levels immediately following the trauma are related to a higher risk of developing PTSD (McFarlane et al., 1997; Delahanty et al., 2000), and to prior trauma (Resnick et al., 1995; Delahanty et al., 2003a). These findings support the hypothesis that a highly sensitized hypothalamic-pituitary-adrenal (HPA) axis may constitute a risk factor for developing the disorder following a traumatic event (Yehuda, 2003). However, studies investigating cortisol levels in chronic PTSD have yielded mixed results (Lemieux and Coe, 1995; Rasmusson et al., 2003; Yehuda, 2003). In addition, studies with children have shown a different pattern of results with high levels of cortisol assessed shortly after a trauma predicting later psychopathology (Delahanty et al., 2005).

Cardiovascular arousal in response to the trauma has also been linked to the development of PTSD. Results of five independent studies showed that high resting heart rates assessed in trauma survivors upon arrival at an emergency department (Shalev et al., 1998; Kassam-Adams et al., 2005; Zatzick et al., 2005; Kuhn et al., 2006) or on the day of discharge from hospital (on average 5 days post-trauma) (Bryant et al., 2000, 2003) significantly predicted the development of PTSD. However, two further studies have shown the opposite pattern of results or null findings (Blanchard et al., 2002; Buckley et al., 2004).

Cognitive and emotional psychological responses to the trauma have repeatedly been shown to be predictive of PTSD, but it remains unclear how they relate to biological risk factors. Psychological predictors identified in earlier research include a history of emotional problems, perceived life threat and strong negative emotions during the event (Ozer et al., 2003) and problematic cognitive processing, in particular, peritraumatic dissociation (Ozer et al., 2003) and data-driven processing (Halligan et al., 2003). The latter is characterized by the predominant processing of sensory impressions (as opposed to the meaning of the situation), which is thought to make subsequent reexperiencing symptoms likely (Ehlers and Clark, 2000).

Although theoretical models make assumptions regarding the relationship between psychological and psychophysiological aspects of the acute trauma response, research studies have as yet not given much attention to this relationship (see Kuhn et al., 2006). Findings from two studies suggest that greater heart rate in the emergency room is related higher levels of peritraumatic distress (Shalev et al., 1998; Kuhn et al., 2006). Kuhn et al. (2006) also found that greater heart rate in the peritraumatic phase is related to greater peritraumatic dissociation, whereas two earlier studies had not found a significant relationship between these variables (Shalev et al., 1998; Delahanty et al., 2003b).

The aim of this prospective longitudinal study was to replicate and extend earlier findings regarding the predictive power of characteristics of the acute response to trauma for the development of PTSD. Psychological and psychobiological predictors identified in earlier studies were assessed in injured MVA survivors within 9 h of their accident (*M* = 3.7 h). Specifically, we aimed to

(1) replicate earlier findings that PTSD symptom severities can be predicted by levels of cortisol and heart rate assessed shortly after the event, and by characteristics of the psychological response to the trauma; and
(2) extend earlier findings (a) by assessing all predictors within the first 9 h post-trauma before post-traumatic stress symptoms have developed and (b) by investigating the relationship between psychological and psychobiological responses to the trauma.

## 2 Method

### 2.1 Participants

The sample comprised 53 injured motor vehicle accident (MVA) survivors. Table 1 shows demographics and accident characteristics. All participants fulfilled the DSM-IV PTSD criterion A (experience of a traumatic event) according to the SCID (First et al., 1996). In addition, results of the Structured Clinical Interview for DSM-IV (SCID) showed that at 6 months follow-up, five participants (9.4%) met criteria for PTSD, two of whom also met criteria for a current major depressive episode. None of the participants met criteria for a current depressive episode without PTSD.

Participants were approached by a researcher while attending the emergency department of a metropolitan hospital less than 12 h after a MVA. Inclusion criteria were: injury in a MVA as a driver, passenger, motorcyclist, or cyclist; age between 18 and 65. Exclusion criteria were: left before receiving medical treatment; very mild injuries (triage category ‘blue’); attended the emergency department more than 12 h after the accident; currently psychotic or suicidal; insufficient command of English to conduct interview; Glasgow Coma Scale (GCS; Teasdale and Jennett, 1974) score of less than 14. After the purpose and procedure of the study had been explained, written informed consent was obtained from the participants. A total of 115 MVA survivors meeting inclusion criteria were approached during a period of 19 months. Of the 74 survivors who were initially interested, 21 did not proceed, and 53 (72%) participated and attended all assessments. Participants who did vs. did not participate did not differ in sex, *Chi*^2^(1, *N* = 74) = 0.05, *P* = 0.82, age, *t*(72) = 0.06, *P* = 0.95, ethnic background, *Chi*^2^(2, *N* = 74) = 0.55, *P* = 0.76, type of MVA, *Chi*^2^(4, *N* = 74) = 2.93, *P* = 0.57, or triage category, *Chi*^2^(2, *N* = 61) = 0.12, *P* = 0.73.

In addition, the final sample of this study (*N* = 53) was compared to a random sample of attenders at the same hospital (*N* = 223). The two samples did not differ in terms of age, *t*(86.49) = − 0.65, *P* = 0.52, ethnic background, *Chi*^2^(2, *N* = 276) = 3.67, *P* = 0.16, or triage category, *Chi*^2^(4, *N* = 276) = 1.57, *P* = 0.82. There was a trend for a difference in sex, with the current sample having a higher percentage of male participants than the random sample, 74% vs. 61%, *Chi*^2^(1, *N* = 274), *P* = 0.09.

### 2.2 Questionnaire and interview measures

#### 2.2.1 Diagnoses of PTSD and depression

Presence of PTSD and depression at 6-month follow-up was assessed using the *Structured Clinical Interview for DSM-IV* (SCID; First et al., 1996). Interrater-reliabilities were high (PTSD: *κ* = 0.82; major depression: *κ* = 1).

#### 2.2.2 Symptom severity measures

PTSD symptom severity was assessed using the *Posttraumatic Diagnostic Scale* (PDS; Foa et al., 1997; *α* in this study = 0.91), a validated and widely used self-report measure of PTSD symptom severity. The *Beck Depression Inventory* (BDI; Beck et al., 1979; *α* = 0.87), a standardized questionnaire of established reliability and validity, assessed the severity of depressive symptoms.

#### 2.2.3 Demographics and accident characteristics

An interview designed for this study was used to gather demographic information as well as information about the accident from the participants.

#### 2.2.4 Injury severity

Injury severity was measured with the *Injury Severity Score* (ISS) (Baker et al., 1974). A trained research nurse specializing in emergency medicine coded the injuries on the basis of the hospital notes.

#### 2.2.5 Past emotional problems

The presence vs. absence of past emotional problems was assessed with the SCID (past major depression, past PTSD) as well as a self-report questionnaire enquiring about any past treatment for emotional problems or substance abuse.

#### 2.2.6 Past traumas

The *Trauma History Interview* was used to assess whether participants had experienced a trauma prior to the MVA. The interview was developed in an earlier study (Ehring et al., 2006) and is based on similar trauma checklists (Blake et al., 1995; Foa et al., 1997). The interview determined the number of past traumatic experiences fulfilling the DSM-IV stressor criteria.

*Perceived life threat during the accident* was assessed by asking participants to rate, on a scale from 0 (not at all) to 4 (very strongly), how much they thought they were going to die during the accident.

*Negative emotions during the MVA* were assessed with the *Peritraumatic Emotions Questionnaire* (Halligan et al., 2003) that asks participants to rate the extent to which they experienced each of 15 different negative emotions during the accident and until help arrived. This questionnaire has been shown to have good internal consistency and to predict PTSD symptoms following trauma (Halligan et al., 2003). In a principal component analysis in an earlier study on MVA survivors, the four subscales ‘fear’ (5 items; e.g., *terrified, alarmed*; *α* in this study = 0.85), helplessness (4 items; e.g., *helpless*, *out of control over what happened*; *α* = 0.79), guilt/shame (3 items; e.g., *guilty*, *ashamed*; *α* = 0.75) and anger (2 items; *angry*, *furious*; *α* = 0.95) were identified (Ehring et al., 2006).

*Cognitive processing during the MVA* was assessed with the *Processing Questionnaire* (Halligan et al., 2003). The questionnaire measures aspects of cognitive processing during the accident, namely data-driven processing (8 items, e.g. *My mind was fully occupied with what I felt, saw, heard and smelled*; *α* = 0.86) and dissociation (9 items, e.g. *I felt emotionally numb*; *α* = 0.91). The measure was developed in a series of studies (Halligan et al., 2002, 2003; Murray et al., 2002), and showed good reliability and validity in predicting intrusive memories and PTSD.

### 2.3 Cortisol assessment

Salivary cortisol samples were obtained using the Salivette device (Sarstedt, Leicester, UK) containing an untreated cotton swab. The first saliva sample was collected during the assessment in the emergency department; thus, these samples were collected at different times of the day (between 10 am and 9:30 pm). In order to control for the diurnal variation of cortisol levels, time of the day was statistically controlled for in all analyses that included cortisol levels assessed at hospital. Participants collected two additional samples on the day following their accident and at 6-month follow-up at 8 am and 10 pm. One morning and one evening sample were chosen in order to allow an assessment of peak and nadir levels of the diurnal cortisol rhythm. Identical times have been used in earlier studies investigating cortisol alterations in trauma survivors (e.g., Aardal-Eriksson et al., 2001; Rohleder et al., 2004). Participants were instructed to refrain from smoking, eating, or drinking anything but water for at least 60 min before saliva collection and to record the exact date and time as well as current levels of pain (assessed on a 0 to 10 visual analogue scale) on the data form. The Salivette tubes were kept in freezers in participants' homes until the 2-week follow-up assessment. After delivery to the laboratory, samples were then stored at − 40 °C until analyzed.

Participants were excluded from the cortisol analyses if they worked night shifts, if they used corticosteroid inhalers, if they had consumed a heavy meal shortly before taking the samples or if they had taken the samples outside of the times indicated. In addition, some participants failed to provide samples at some assessments. Samples were included in the analyses from 41 participants in the emergency room (77%) and from 35 participants (66%) for the morning sample as well as 33 participants (62%) for the evening sample on the day following the accident. At 6-month follow-up, the numbers were 26 (49%) for the morning samples and 25 (47%) for the evening samples. At none of the assessments, did participants included in the cortisol analyses differed from those excluded in age, all |*t*(51)| < 1.39, all *P*'s > 0.17, sex, all *Chi*^2^(1, *N* = 53) < .77, all *P*'s > 0.38, type of road user during accident, all *Chi*^2^(3, *N* = 53) < 3.83, all *P*'s > 0.28, injury severity scores, all |*t*(51)| < 0.66, all *P*'s > .51, or triage category, all *Chi*^2^(2, *N* = 53) < 1.44, all *P*'s > 0.49.

Samples were first thawed and centrifuged at 3000 rpm for 10 min. The free cortisol levels in saliva were then measured using a time-resolved fluoroimmunoassay as described elsewhere (Pariante et al., 2002), except that the rabbit cortisol antibody (product no. 2330-5105, batch 21051565; Biogenesis, Poole, Dorset, UK) and the europium-labeled cortisol were diluted 1/4500 and 1/65, respectively, in assay buffer before use.

A short interview was conducted to establish whether participants worked night shifts, were using corticosteroid inhalers or any medication as well as alcohol, drug and nicotine consumption during the past week and current levels of pain (assessed on a 0 to 10 visual analogue scale).

### 2.4 Heart rate and blood pressure

Information about participants' heart rate and blood pressure upon arrival at the emergency department was taken from the hospital notes. In addition, a two minute assessment of resting heart rate was conducted during the assessment session. Inter-beat intervals were recorded continuously for 2 min using a Polar S810 heart rate monitor (Polar Electro, Vantaa, Finland), consisting of a chest band and a wrist receiver. The device has been shown to reliably assess inter-beat intervals during rest as well as activity (Seaward et al., 1990; Wajciechowski et al., 1991; Laukkanen and Virtanen, 1998). Recorded data were subsequently examined using Polar Precision Performance Software version 3.0. Artifacts in the data due to misdetected R-waves in the electrocardiogram were easily discernible as outliers from the average HR curve, and were deleted and interpolated prior to averaging using the software error correction facility (artifacts were less than 0.5% of the data for all participants). Participants were instructed to lie quietly, not to speak and to keep their eyes open. During the heart rate assessment, the interviewer was seated at a distance of approximately 2 m from the participants, kept his head lowered and avoided eye contact. Heart rate data were available for 45 participants (84.9%). None of the participants were taking beta-blockers or other hypertensive medication.

### 2.5 Additional variables

A number of additional variables were assessed at 2 weeks after the MVA and are reported elsewhere (Ehring et al., 2008).

### 2.6 Procedure

The study was approved by the local research ethics committees.

After written informed consent had been obtained, demographic information and information about the accident was collected. Participants then filled in the *Peritraumatic Emotions Questionnaire* and the *Processing Questionnaire*. After that, the first saliva sample was collected and participants were interviewed about possible confounding factors. Next, the resting heart rate was measured. At the end of the assessment, participants received an envelope with the material for taking the two saliva samples on the day following the accident. Participants were again contacted 2 weeks and 6 months later for follow-up assessments.

### 2.7 Statistical analyses

As only five participants met criteria for PTSD at 6-month follow-up, no group differences were computed because of lack of power for this type of analysis. Instead, the hypotheses were tested by computing Pearson product-moment correlation coefficients between predictor variables and symptom severity measures as well as between the different groups of predictor variables. In addition, partial correlations were computed in order to statistically control for possible confounding variables (e.g., time of day at first assessment). For categorical predictor variables, *t* tests and analyses of variance (ANOVAs) were computed. Mean cortisol levels as well as a number of questionnaire scores were skewed and therefore transformed to normal via log-transformation prior to analyses.

All statistical tests were two-tailed.

## 3 Results

### 3.1 Prediction of symptom severities by demographic and accident variables

Symptom levels of PTSD and depression were not significantly associated with sex (all |*t*| < 2; all *P*'s > 0.05), ethnic background (all |*F*| < 2; all *P*'s > 0.10), injury severity scores (all |*r*| < .28; all *P*'s > 0.09) or type of road use during the accident (driver, passenger, bicyclist motorcyclist; all *F*'s < 1; all *P*'s > 0.30).

### 3.2 Prediction of PTSD and depressive symptom severities by predictor variables

Correlations between the predictor variables and symptom levels of PTSD and depression are shown in Table 2. Predictors of high PTSD symptom severities at 6 months were: low levels of cortisol (time of day was controlled statistically) and low diastolic blood pressure assessed in the emergency room, past emotional problems, dissociation, and data-driven processing during the event. Predictors of depressive symptoms were: low cortisol levels in the emergency department and greater number of past traumatic experiences. No significant correlations with symptom severities were found for heart rate and systolic blood pressure or cortisol 1 day post-MVA. High evening levels of cortisol at 6 months were, however, associated with more severe PTSD and depressive symptoms. Pain severity at 6 month correlated significantly with both evening cortisol levels (*r* = 0.69) and symptom severities (PTSD: *r* = 0.37, depression: *r* = 0.41). When pain severity was statistically controlled with partial correlations, the relationship between evening cortisol and symptom severity was no longer significant (PTSD *r* = 0.21, depression *r* = 0.20).

### 3.3 Relationships between predictor variables

For women, low cortisol levels in the emergency room were related to past emotional problems (ANCOVA with ‘time of the day’ as covariate: *F*(1, 8) = 26.93, *P* < 0.01), and past traumatic events (*partial r* = − 0.71, *P* < 0.05). No relationship was found for men, *F*(1, 22) = 0.24, *P* = 0.63; and *partial r* = 0.00, *P* = 0.99, respectively. In the total sample, low cortisol levels in the emergency room correlated with shame/guilt, *r* = − 0.39, *P* < 0.05, and data-driven processing during the accident, *r* = − 0.33, *P* < 0.05.

Blood pressure and heart rate in the emergency room were not significantly correlated with cortisol levels or any variables of cognitive and emotional processing during the accident (all |*r*| < 0.28, all *P* > 0.12).

### 3.4 Regression analyses

To test which variables best predicted symptom levels of PTSD and depression at 6-month follow-up, two linear regression analyses were conducted. All predictor variables showing significant correlations with the symptom severity measures at follow-up were included in the analyses as predictor variables. A combined score of dissociation and data-driven processing was computed to represent cognitive processing during the accident and residualized cortisol levels taken in the emergency room controlled for time of the day were used. When predicting PTSD symptom severity at follow-up, the combined cognitive processing score (*B* = 0.04, *SE B* = 0.02, *β* = 0.50, *P* < 0.05) and the 'past emotional problems' score (*B* =1.22, *SE B* = 0.46, *β* = 0.44, *P* < 0.05) were significant predictors (*R*^2^ = 0.60), and ‘cortisol levels at the emergency room’ (*B* = 0.10, *SE B* = 0.35, *β* = 0.05, *P* = 0.77) and ‘diastolic blood pressure’ (*B* = − 0.02, *SE B* = 0.02, *β* *=* − 0.18, *P* = 0.29) did not predict unique variance. When predicting depression severity at 6 months, ‘past emotional problems’ (*B* = 1.29, *SE B* = 0.40, *β* = 0.46, *P* < 0.01) and the residualized cortisol scores at the emergency room (*B* = − 0.61, *SE B* = 0.29, *β* = − 0.30, *P* < 0.05) explained a significant proportion of the variance (*R*^2^ = 0.53), whereas ‘number of past traumas’ did not (*B* = 0.15, *SE B* = 0.08, *β* = 0.27, *P* = 0.07).

## 4 Discussion

The aim of this study was to replicate and extend earlier findings regarding the prediction of PTSD and depressive symptom severities by the acute psychobiological and psychological trauma response. Predictors were assessed within 9 h following injury in a motor vehicle accident.

In line with preliminary earlier findings (McFarlane et al., 1997; Delahanty et al., 2000) *low* levels of cortisol assessed within the first hours after the accident predicted PTSD symptom severity at 6 months. These results are consistent with the hypothesis that an increased sensitivity of the negative feedback system of the HPA axis resulting in hypocorticolism might constitute a risk factor for PTSD (Yehuda, 2003). In line with previous research (Resnick et al., 1995; Delahanty et al., 2003a), we also found a correlation between low cortisol levels in the emergency room and past traumas and past emotional problems, which points to the possibility that previous traumas may chronically attenuate cortisol reactivity, which in turn may increase the risk of PTSD.

How can the relationship between low cortisol and the development of PTSD symptoms be explained? Nearly all hypotheses put forward to date draw on the idea that an abnormal psychobiological response to the trauma leads to impaired information processing of the event, which in turn causes the memory-related symptoms (especially intrusive re-experiencing) that lie at the core of the disorder. Whereas some authors suggest that low cortisol might directly interfere with the elaboration of the trauma memory (Schelling et al., 2004), others hypothesize that the relationship between low cortisol and PTSD could be mediated by an excessively high response of the sympathetic nervous system that is not appropriately modulated by the HPA axis (Yehuda and Harvey, 1997). Our study found a relationship between compromised information processing during the trauma, namely greater data-driven processing, and low cortisol. High levels of data-driven processing refer to a predominant encoding of sensory impressions during the trauma and insufficient processing of the meaning of the situation. This is thought to lead to poorly elaborated autobiographical memory traces of the trauma, and poor inhibition of unwanted cue-driven intrusive memories (Ehlers and Clark, 2000). Low cortisol levels in hospital were also related to high levels of shame and guilt during the trauma, which mirrors earlier findings regarding a relationship between low cortisol and shame, guilt as well as disengagement coping strategies (Mason et al., 2001).

In line with earlier findings (Ozer et al., 2003), a history of emotional problems, perceived life threat, negative emotions and compromised information processing during the trauma (dissociation and data-driven processing) correlated significantly with initial symptom levels of PTSD, but not depression. At 6-month follow-up, only a history of emotional problems and compromised information processing remained significant predictors of PTSD symptom levels.

The study also showed some unexpected findings that need to be interpreted with caution. In contrast to McFarlane et al. (1997), who found that high cortisol in the emergency room predicted subsequent depression, this study found a correlation between low cortisol and subsequent depressive symptoms. The reasons for this discrepancy remain unclear. Furthermore, the cortisol assessments on the day following the MVA and the morning 8 am value at 6-month follow-up showed no association with symptom levels. This finding is discrepant from the majority of earlier studies that found low cortisol in chronic PTSD (Yehuda, 2003), albeit usually with PTSD of greater chronicity or following repeated trauma, but is in line with a small number of studies showing negative results (Rasmusson et al., 2003). The only significant effect was an association between *high* evening levels of cortisol at 6-month follow-up with more severe PTSD and depressive symptoms. This finding remains to be interpreted with caution, but is consistent with a parallel study of assault survivors (Kleim, 2006). High evening cortisol levels correlated with greater pain severity at 6 months, and the relationship with PTSD and depression severity was no longer significant when pain severity was partialled out. These results suggest that cortisol levels may be influenced by different stressors in different ways. Pain, which tends to be worse in the evenings, may be a chronic stressor that increases cortisol levels, possibly masking any effects of trauma exposure. Alternatively, high evening cortisol could be a marker of a flattened cortisol profile that has been found to be associated with a number of stress-related disorders (Fries et al., 2005). Future research should use a more comprehensive assessment of cortisol levels and rigorously control for possible confounding factors, especially levels of pain.

Heart rate in the emergency room was not related to symptom levels of PTSD or depression in this study. Like some other studies (Blanchard et al., 2002; Buckley et al., 2004), our study thus failed to replicate the relationship between high heart rates in the immediate aftermath of trauma and subsequent PTSD (Shalev et al., 1998; Bryant et al., 2000; Zatzick et al., 2005; Kuhn et al., 2006). It is possible that differences in trauma type and procedural differences contributed to the different results. However, in this study levels of diastolic blood pressure were significantly negatively correlated with PTSD symptom severity at 2 weeks and 6 months. Taken together, the results from the studies looking at cardiovascular arousal in the acute trauma phase are inconsistent. Future research will need to investigate context as well as methodological variables that may influence the relationship between cardiovascular arousal in the acute trauma phase and the development of PTSD (for a detailed discussion, see Kuhn et al., 2006).

A number of limitations of the study are noteworthy. Firstly, the sample size was rather small, resulting in decreased power for the statistical analyses. In addition, because of the small sample size no elaborate statistical approaches for longitudinal data, e.g. mixed effect models, could be computed. However, the size of the sample is comparable to or exceeds that of earlier studies of the acute trauma response (Resnick et al., 1995; McFarlane et al., 1997). Secondly, only few participants in our sample developed syndromal PTSD. Therefore, no differences between diagnostic groups could be computed. Future studies should aim to test larger samples of trauma survivors in order to increase the number of participants who meet the full disorder criteria. Nevertheless, as earlier studies have found subthreshold levels of PTSD to be associated with considerable impairment (e.g., Schützwohl and Maercker, 1999), the investigation of predictors of PTSD and depressive symptoms, as in this study, appears relevant and of interest. Thirdly, assessing psychobiological factors in the acute trauma phase makes it difficult to control for confounding factors such as the type and impact of injuries, pain, time of the day or medication received in the emergency room. In this study, some of these variables were statistically controlled in order to eliminate their influence on the hypotheses studied. Future studies should investigate the influence of these variables on cardiovascular or cortisol levels in more detail. Finally, it is noteworthy that injury severities were low in our sample. Future studies should aim to include participants with a broader range of injury severities in order to test the impact of this variable on the findings.

Despite these limitations, the results support the role of the acute response to trauma in the development and maintenance of PTSD and provide promising preliminary evidence for a meaningful relationship between psychobiological and psychological factors in the acute trauma phase.

## Figures and Tables

**Table 1:** Demographics, accident characteristics and symptom severities

Variable		*N* or *M*	% or SD
Sex	Male	39	73.6%
	Female	14	26.4%

Age (years)		34.02	8.65

Ethnic background	Caucasian	40	75.5%
	Black	8	15.1%
	Other	5	9.4%

Marital status^1^	Single	35	66%
	Married	11	20.8%
	Divorced/separated	6	11.3%

Education (years)		14.64	4.06

Employment status^2^	Working	41	77.4%
	Student	4	7.5%
	Not working	6	11.3%

Type of road user during accident	Driver	14	26.4%
	Passenger	4	7.5%
	Motorcyclist	25	47.2%
	Bicyclist	10	18.9%

Hours elapsed between MVA and first assessment		3.71	1.74

Injury severity score		1.83	1.71

Posttraumatic Diagnostic Scale (PDS)	2 weeks	10.04	10.26
	6 months	6.12	7.32

Beck Depression Inventory (BDI)	2 weeks	6.56	6.56
	6 months	6.53	8.73

^1^*n* = 52; ^2^*n* = 51.

**Table 2:** Correlations between predictor variables and symptom severities

Predictor variables		PTSD symptom severities (PDS)		Depressive symptom severities (BDI)	
	*M* (*SD*) or *N* (%)	2 weeks	6 months	2 weeks	6 months
*Cortisol (nmol/l)*^1^					
in emergency room	12.18 (10.51)	− 0.33^⁎^^2^	− 0.33^⁎^^2^	− 0.05^2^	− 0.45^⁎⁎^^2^
day after MVA 8 am	12.45 (4.81)	0.21	0.12	− 0.25	− 0.01
day after MVA 10 pm	4.40 (3.89)	0.17	0.17	0.00	0.06
6-month FU 8 am	13.19 (5.32)	N/I	− 0.10	N/I	− 0.07
6-month FU 10 pm	5.44 (8.83)	N/I	0.40^⁎^	N/I	0.42^⁎^

*Cardio-vascular variables*					
Heart rate (bpm) taken from hospital notes	74.97 (15.70)	− 0.18	− 0.17	0.29	0.11
Mean resting heart rate (bpm) during assessment	72.93 (11.16)	− 0.22	− 0.15	0.14	0.06
Systolic blood pressure (mmHg)	125.94 (14.71)	− 0.25	− 0.20	0.14	− 0.03
Diastolic blood pressure (mmHg)	72 (14.64)	− 0.42^⁎^	− 0.37^⁎^	− 0.08	− 0.18

*Questionnaire results*					
Past emotional problems^3^	Yes: 25 (47.2%) No: 28 (52.8%)	0.25	0.36^⁎^	0.20	0.56^⁎⁎^
Number of past traumas	2.40 (2.27)	0.11	0.13	0.10	0.33^⁎^
Pain in emergency room	4.03 (2.54)	0.36^⁎^	0.27	0.44^⁎⁎^	0.26
Pain severity at 6 months	.33 (1.23)	N/I	0.31	N/I	0.41^⁎^
Perceived threat to life	2.89 (2.81)	.34^⁎^	0.16	− 0.01	0.16
Fear	9.39 (5.16)	0.42^⁎⁎^	0.18	− 0.01	0.06
Helplessness	8.00 (4.77)	0.48^⁎⁎^	0.19	0.09	0.06
Guilt/shame	1.18 (1.96)	0.37^⁎⁎^	0.24	0.01	0.17
Anger	3.80 (3.02)	0.24	− 0.04	0.16	− 0.08
Dissociation	6.83 (7.49)	0.48^⁎⁎^	0.29^⁎^	0.01	0.18
Data-driven processing	8.95 (7.12)	0.54^⁎⁎^	0.32^⁎^	− 0.02	0.27

MVA: motor vehicle accident; FU: follow-up; ^⁎^*P* < 0.05; ^⁎⁎^*P* < 0.01; N/I: not of interest for the hypotheses tested. ^1^Cortisol levels were not significantly correlated with the Body Mass Index, injury severity, cigarettes smoked per day, alcohol units consumed in past week, drug use in past week and anti-depressant medication taken or time elapsed since the accident. ^2^Time of day partialled out. ^3^Correlations in this row are point-biserial correlation coefficients.
